# Life History and Reproductive Parameters in an Increasingly Abundant Killer Whale Population

**DOI:** 10.1002/ece3.74185

**Published:** 2026-08-19

**Authors:** Chloe Kotik, Thomas Doniol‐Valcroze, Jared R. Towers, Lara Horstmann

**Affiliations:** ^1^ College of Fisheries and Ocean Sciences University of Alaska Fairbanks Fairbanks Alaska USA; ^2^ Pacific Biological Station Fisheries and Oceans Canada Nanaimo British Columbia Canada; ^3^ Bay Cetology Alert Bay British Columbia Canada

## Abstract

Life history parameterization is foundational to population monitoring, particularly for long‐lived species under targeted management schema. In the eastern North Pacific, multidecadal photographic identification studies have facilitated the estimation of life history parameters for several distinct populations of fish‐eating “Resident” killer whales ( *Orcinus orca ater* ). However, to date, comparable estimates have not been generated for the increasingly abundant West Coast Transient (WCT) population of mammal‐eating “Bigg's” killer whales (*Orcinus orca rectipinnus*), despite their designation as “Threatened” under the Canadian Species at Risk Act. We used over 50 years of photographic identification data to estimate life history and reproductive parameters for WCT for the first time and compared our estimates to those previously published for sympatric Resident killer whale populations. Several aspects of WCT life history were consistent with Resident killer whales: mean age of first successful recruitment in WCT was 15.2 years (SE = 0.4), while average WCT calving interval was 4.88 years (SE = 0.13), yielding average annual fecundity of 0.20 calves per mature WCT female per year. However, WCT showed potential evidence of decreasing age at first recruitment (regression slope = −0.07, *p* = 0.08), were the only population examined to exhibit decreasing calving intervals over the course of their study period (1972–2024), and had the lowest calf mortality of any killer whale population assessed in this study. While shared evolutionary lineage conserves many aspects of life history among killer whales, these potential differences may be associated with ongoing WCT population growth over the last 5 decades and could reflect ecological conditions, including increasing prey abundance, within their core range.

## Graphical abstract

In the coastal waters of the eastern North Pacific, multiple discrete populations of killer whales exhibit distinct patterns in demography and abundance that are partially captured by population vital rates. We used 50 years of photographic identification data to estimate parameters relevant to maturation, fecundity, and mortality for a unique population of mammal‐eating killer whales and compared our results with previously published estimates for three populations of fish‐eating killer whales. While some aspects of life history were conserved across populations, detected differences in certain vital rates likely describe underlying evolutionary and ecological conditions impacting population growth.

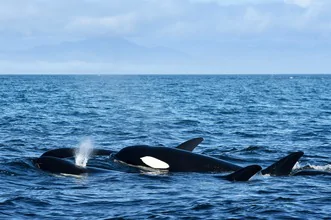

## 1 Introduction

Life history parameterization is a critical component of ecological research that can yield valuable insight into population dynamics, demographics, and abundance trends (Skalski et al. 2005). When contextualized by environmental and ecological variation, parameters related to survival and recruitment can shed light on underlying sources of change at the population level; these parameters can also be used to examine whether species are meeting management or conservation goals (Murray et al. 2021). However, the estimation of life history parameters can be challenging for wild populations, particularly for long‐lived, k‐selected species such as cetaceans. When not derived from measurements of stranded, bycaught, hunted, or captive individuals, life history parameters of cetaceans are typically estimated via longitudinal studies that require identification and monitoring at the individual level (Clapham and Mayo 1987; Mann et al. 2000; Wells et al. 2025).

**FIGURE 1:**
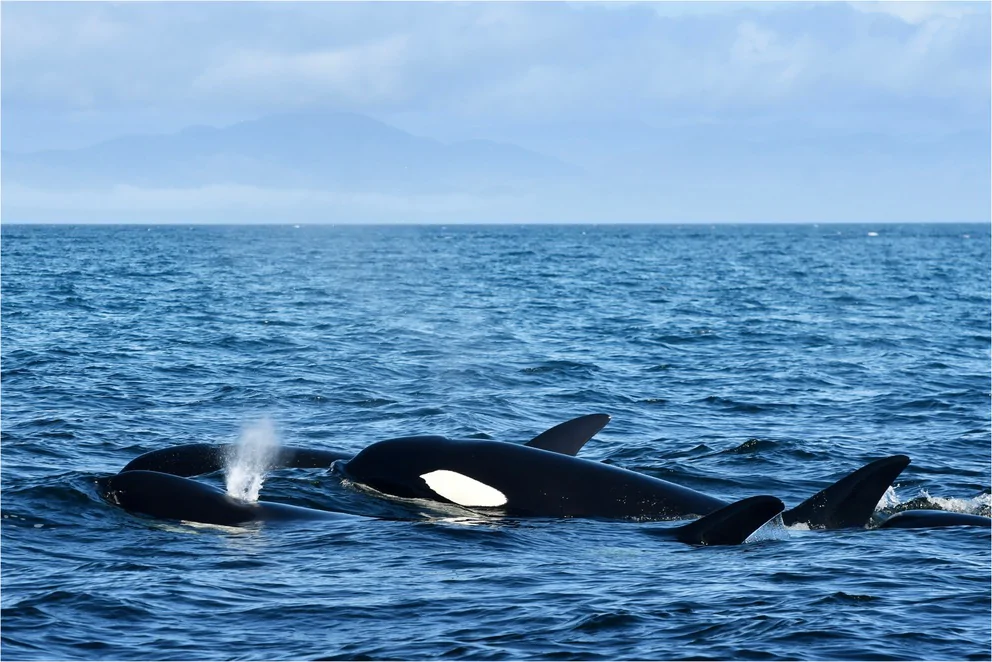
Three West Coast Transient killer whales surfacing in Queen Charlotte Strait, British Columbia, Canada. Photo taken by Chloe Kotik under Canadian research license MML‐042.

The West Coast Transient (WCT) population of mammal‐eating Bigg's killer whales (*Orcinus orca rectipinnus*) has been monitored via photographic identification in the coastal eastern North Pacific for over 50 years (Figure 1; Bigg 1982; Ford and Ellis 1999; Towers et al. 2019). In that time, regular population updates have documented consistent annual growth for a coastal subset of individuals commonly found in British Columbia, Canada (Ford et al. 2013; Towers et al. 2019). This subset included ~385 individuals at the end of 2024 (Towers et al. 2025). The underlying forces supporting population growth in the coastal subset have not been extensively studied, but have been linked to the regional recovery of several important prey populations following the implementation of marine mammal protections in the United States and Canada in the 1970s (Elliser and Hall 2021; Jefferson et al. 2023; Laake et al. 2018; Majewski et al. 2024; Pearson et al. 2025; Shields et al. 2018; Tucker et al. 2025).

While the abundance of the coastal subset has increased in the decades since federal protections were implemented, the broader WCT population, which ranges from California to Alaska, is still considered vulnerable to perturbation from anthropogenic contamination, physical and acoustic disturbance, and changes to prey availability or quality (Ford et al. 2007; Lee et al. 2022; Murray et al. 2025; Ross et al. 2000). Consequently, it has been designated “Threatened” under the Canadian Species at Risk Act since 2001 (DFO 2009). Effective monitoring and management of this population would be bolstered by quantitative estimates of life history parameters, particularly those relevant to demographic and ecological underpinnings of population growth. However, not all life history parameters have been estimated at the subspecies or population level for mammal‐eating killer whales. Instead, their life history has typically been assumed to be similar to fish‐eating Resident killer whales in the region, despite differences in relevant ecological conditions (e.g., prey abundance) and some evidence that the two forms, currently listed as subspecies of *O. orca* , may constitute separate species (Morin et al. 2024).

WCT overlap with three distinct populations of Resident killer whales in coastal waters: Southern Resident killer whales (SRKW) ranging from northern California to southeastern Alaska (Carretta et al. 2023; Center for Whale Research 2025; Fisheries and Oceans Canada 2007), Northern Resident killer whales (NRKW) ranging from northern Washington to southeastern Alaska (Towers et al. 2020), and Southern Alaska Resident killer whales (SARKW) ranging between northern British Columbia, Canada, and Kodiak, AK (Matkin et al. 2014; Myers et al. 2021). WCT are morphologically, behaviorally, and genetically distinct from Resident killer whales (Baird and Dill 1995; Baird and Whitehead 2000; Bigg 1982; Deecke et al. 2005; Emmons et al. 2019; Foote and Morin 2016; Kotik et al. 2022; Riesch and Deecke 2011); population dynamics and abundances have also varied among all four populations (Matkin et al. 2014; Nielsen et al. 2021; Towers et al. 2019; Williams et al. 2024). SRKW, which are listed as Endangered in the United States and Canada, number just ~74 individuals and have been in decline since at least the mid‐1990s (Center for Whale Research 2025). NRKW, listed as Threatened in Canada, experienced a temporary decline in the late 1990s and early 2000s, but have been consistently increasing in abundance since (Towers et al. 2020). With the exception of one pod that was exposed to the Exxon Valdez oil spill, SARKW (unlisted in the United States and Canada) are also thought to be increasing in abundance (Matkin et al. 2014, 2018). Life history parameters, including age of maturation, calving rates, typical calving intervals, and age‐specific mortality, have been previously estimated for SRKW, NRKW, and SARKW, but not WCT (Matkin et al. 2014; Murray et al. 2021; Olesiuk et al. 2005; Vélez‐Espino et al. 2014). These estimates can provide valuable insight towards understanding demographic variation, population dynamics, and responses to anthropogenic stressors and environmental conditions. Given the established divergence between WCT and Resident killer whale populations, life history parameters and population vital rates of WCT should be evaluated separately.

This study leveraged over five decades of photo‐identification data to quantify the life history of WCT independently of other killer whale populations in the region. We focused on the parameters most relevant to population growth (i.e., maturation, fecundity, and mortality) and, where possible, compared our estimates for WCT with those published for SRKW, NRKW, and SARKW to contextualize demographic patterns with evolutionary divergence, investigate the extent of population‐ and subspecies‐level variation, and support additional inquiries into the ecological mechanisms of population growth.

## 2 Methods

### 2.1 Data Collection and Processing

Demographic records for WCT were obtained from dedicated and opportunistic photo‐identification encounters from 1972 to 2024. WCT were primarily photographed in coastal waters of southeastern Alaska, British Columbia, and northern Washington, but were also photographed in Oregon and California (Towers et al. 2019, 2025). Individual whales were identified by the shape of the dorsal fin and by congenital and acquired pigmentation patterns on saddle and eye patches, including nicks and scars. These markings are largely persistent, enabling the re‐identification of individuals over years or decades (Bigg 1982). Photographed encounters were used to maintain annual (1972–2018) and quarterly (2019–2024) records of an individual's presence and status (alive/dead/unknown). Records were maintained in a central data repository by Bay Cetology for the Department of Fisheries and Oceans Canada. Not every individual was observed each year: in cases where an individual's status in a given year was unknown, positive identifications in subsequent years were used to assess status retroactively.

For whales born during the survey period and first encountered as calves, a best estimate of birth year was conservatively based on appearance at first sighting, including size, coloration, and the presence or absence of latitudinal creases found on the skin of newborn individuals (Ford 2009; Reidenberg and Laitman 2009). Minimum birth year was estimated from the date a calf's mother was last seen without it, while maximum birth year was estimated as the year in which a calf was first seen. The majority of individuals were observed within the first 5 years of life, but females that were born prior to the beginning of the survey (or were physically mature at first documentation) were assigned a maximum birth year 10 years prior to the year in which they were first observed (Towers et al. 2019). Immature or maturing males born prior to the beginning of the survey were assigned a minimum birth year based on the height of the dorsal fin, while fully grown males were assigned a maximum birth year 20 years prior to the year they were first observed (Towers et al. 2019). Maternal relationships were determined by an individual's continued association with a reproductive‐age female. Sex was determined and retrospectively applied based on the observation of secondary sexual characteristics (i.e., sprouting of the dorsal fin in males), the recruitment of a calf, genetic analyses of sampled tissues, or observation of characteristic pigmentation in the ventral region (Towers et al. 2019).

In this study, the death of a missing individual was occasionally confirmed through recovery of an identifiable carcass, but most WCT deaths were estimated based on contrasting patterns of association. Dependent calves (< 3 years old) were declared dead after their family groups were documented without them in a single “complete” encounter (i.e., an encounter where all individuals present were photographed). For older animals, multiple complete encounters with known long‐term associates over a period of months or years were used to judge if the individual was consistently missing and therefore presumed dead (Towers et al. 2019). All deceased individuals were assigned a minimum death year (the year of their last sighting) and a maximum death year based on the above criteria, which were used to estimate minimum, maximum, and mean age at death. In the absence of conditions meeting these criteria, individuals were presumed alive in a given year or quarter even if not directly observed.

While WCT killer whales range from at least southern California to Alaska, most WCT individuals included in our analyses belonged to a subset documented in nearshore British Columbia and adjacent waters (Ford et al. 2007, 2013; Towers et al. 2019, 2025). While their proximity to dense coastal human population centers does render whales in this coastal subset more vulnerable to anthropogenic impacts than WCT found further offshore, coastal subset whales are not otherwise known to be biologically distinct from other WCT killer whales and do not represent a discrete breeding unit. Instead, this subset was defined to enable scientifically meaningful examinations of abundances and population dynamics over multiple decades of monitoring. Subset status has been defined by the length of time since last documentation, total number of encounters, and cumulative number of years documented within the study region, though the exact criteria have shifted slightly over the years to accommodate the growing encounter database and changing survey effort (Ford et al. 2007, 2013; Towers et al. 2019, 2025), resulting in potentially fluid membership over time. While most individuals used to estimate life history parameters in this study have been considered part of the coastal subset, these calculations were not explicitly restricted to those whales, and a handful of individuals were not included in the 2025 coastal subset population update (Table S1, Towers et al. 2025).

### 2.2 Recruitment

Following Olesiuk et al. (2005), female WCT were considered mature upon the recruitment of a viable (surviving for at least 1 year) calf. Using DeMaster (1978), we estimated the mean age of first recruitment (AFR) as follows:

$$f\left(x\right)=\frac{t\left(x\right)}{n\left(x\right)}$$

where *t*(*x*) = the number of females of age *x* that have recruited a calf and survived to age *x* and *n*(*x*) = the total number of females that survived to age *x*. Individuals were included in *t*(*x*) and *n*(*x*) even if they were not directly observed at age *x* (i.e., if they were observed in later years and could be retrospectively confirmed alive at age *x*). This yielded *P*(*x*), the estimated probability of recruiting a calf at age *x*:

$$P\left(x\right)=f\left(x\right)-f\left(x-1\right)$$

AFR was then calculated as follows:

$$\mathrm{AFR}=\sum \left(x\right)P\left(x\right)$$

Variance and 95% confidence intervals were calculated using bootstrap sampling of individual whale documentation histories (as in Matkin et al. 2014). When the age of a female whale was uncertain (i.e., when an individual was already mature when first encountered) or was estimated to be ≥ 10 years at the beginning of the study, we could not assume that her oldest living offspring was her first viable calf. Therefore, the estimation for mean AFR was restricted to females that were born, matured, and gave birth to a viable first calf during the survey period. Females that gave birth to their first calf in 2024 were also excluded from AFR estimates, as a full year after birth was required to determine whether those calves should be considered viably recruited (Table S2).

Gaps in observational records can also contribute to the uncertainty surrounding life history estimates: when whales go undocumented for multiple years, it is possible for calves to be born, survive long enough to be considered viable, and die without being documented. In this study, an observational gap was defined as a calendar year in which an individual was not documented. Mean AFR was calculated separately for a subsample of females (Table S2) with no annual observational gaps between age 10 and first recruitment. While it is still possible for calves to go unobserved when females are seen yearly, an increased frequency of documentation was expected to reduce the possibility of viable calves going undetected and biasing AFR. To facilitate additional cross‐population comparisons, we also estimated mean age of first reproduction (AFRP) in WCT. AFRP was estimated using identical methods to AFR, but was inclusive of all births (i.e., including calves that did not survive to age 1), whereas AFR only included calves that survived to age 1. This included calves born in 2024 whose viability was not yet known (Table S2).

The effect of birth year on AFR, in both the complete sample and subsample, was tested for nonlinearity using generalized additive models (GAMs). In the absence of strong evidence for nonlinearity (e.g., where the *edf* of a smoothed term was penalized to ~1) we subsequently modeled relationships between variables using generalized linear models (GLMs) with a Gaussian error distribution and identity link function (Wood 2017; Zuur et al. 2009). We used *q*–*q* plots to visually inspect residual normality and stepwise selection and small‐sample Akaike information criterion to identify the best candidate model (AICc; Symonds and Moussalli 2011). All candidate models were examined for residual normality, homoscedasticity, and autocorrelation. All analyses were conducted via the *stats* and *mgcv* packages of the R software environment (Wood 2017; R version 4.4.2, R Core Team, 2024, https://www.r‐project.org).

### 2.3 Fecundity

To minimize variability from uncertain age estimates, age‐specific fecundity of female killer whales (*FEC*_*f*(*x*)_) was estimated only for females that were < 10 years old in 1972 (Table S3). *FEC*_*f*(*x*)_ was calculated as the proportion of females of age *x* that gave birth to a viable calf each year:

$${\mathrm{FEC}}_{f\left(x\right)}=\frac{{\mathrm{NC}}_{f\left(x\right)}}{N_{f\left(x\right)}}$$

where *NC*_*f*(*x*)_ = the number of calves born to females of age *x* and *N*_*f*(*x*)_ = the total number of females of age *x*. To enable cross‐population comparisons, we also estimated mean *FEC*_*f*(*x*)_ of WCT by pooling across decadal age classes (20–29, 30–39, 40–49 years) after Olesiuk et al. (2005) and across bins for “young” reproductive females (10–29 years) and “old” reproductive females (30–50 years), defined by Vélez‐Espino et al. (2014). A GAM smooth was fit to WCT fecundity using thin‐plate regression splines and a Tweedie error distribution with a log‐link function via restricted maximum likelihood in *mgcv* (Wood 2017). The Tweedie distribution was selected on the grounds of its suitability for positive, zero‐inflated ecological data similar to those captured by our estimates of age‐specific fecundity (Wood 2017).

The mean annual fecundity rate of WCT was estimated using the reciprocal of the mean calving interval for all viable calves born during the study period (Table S3; Olesiuk et al. 2005). Because calving intervals may be affected by calves that were born and died without being documented, we also estimated mean calving interval and annual fecundity rate from a subsample of calving intervals from female WCT that were documented at least every other year beginning from age 10 (Table S3). Given that killer whales gestate for 15–18 months (Duffield et al. 1995; Robeck et al. 2004), and a viable calf was defined as surviving for at least a year, restricting estimates to females documented at least every other year may reduce the likelihood of unknowingly including females that have given birth to viable but undocumented calves.

Effects of calving year (*CY*) and maternal age at birth (*MAB*) on calving interval (*CI*) were tested for nonlinearity using GAMs. In cases where the *edf* value of a smoothed term was penalized to 1, we subsequently modeled using a series of nested GLMs (i.e., *CI* ~ *CY* + *MAB*; Wood 2017; Zuur et al. 2009) with a Gaussian error distribution and identity link function. We used *q*–*q* plots to visually inspect residual normality and stepwise selection followed by small‐sample Akaike information criterion to identify the best candidate model (AICc, Symonds and Moussalli 2011; Table S4). All candidate models were examined for residual normality, homoscedasticity, and autocorrelation. We also used a random resampling approach to evaluate the sensitivity of our model to temporal changes in survey effort. For each of 1000 iterations, we randomly sampled up to 3 calving intervals per calving year without replacement, fit our best candidate model, and recorded the significance of calving year and maternal age at birth as linear predictors of calving interval length at α = 0.05. We then calculated the proportion of simulations for which each predictor variable met the threshold for significance.

### 2.4 Mortality

As life expectancy and survival trajectories for Bigg's killer whales have been described elsewhere (Nielsen et al. 2021), we limited analyses of WCT mortality to the first 20 years of life to compare with sympatric Resident killer whale populations. Annual mortality rate at age *x* (*MR*(*x*)) was calculated for killer whales born during the study period as follows:

$$\mathrm{MR}\left(x\right)=1-\mathrm{SR}\left(x\right)=\frac{D\left(x\right)}{N\left(x\right)}$$

where *SR*(*x*) = annual survival rate of animals aged *x*, *D*(*x*) = the number of animals aged *x* that died before reaching age *x* + 1, and *N*(*x*) = the number of animals in the study population aged *x* that were monitored to age *x* + 1. Mortality of individuals with uncertain death years was amortized across all the years in which they may have died (i.e., evenly divided across all years between minimum and maximum death year estimates). Annual mortality rate was calculated by pooling data across the progressively widening age classes used by Murray et al. (2021) and Vélez‐Espino et al. (2014): 0–1, 1–2, 2–5, 5–10, and 10–16 years. Standard errors and confidence intervals of *MR*(*x*) were calculated using bootstrap sampling of animal‐years. Because sex is typically not known for juvenile killer whales, estimates were pooled across sex.

## 3 Results

### 3.1 Encounters

**FIGURE 2:**
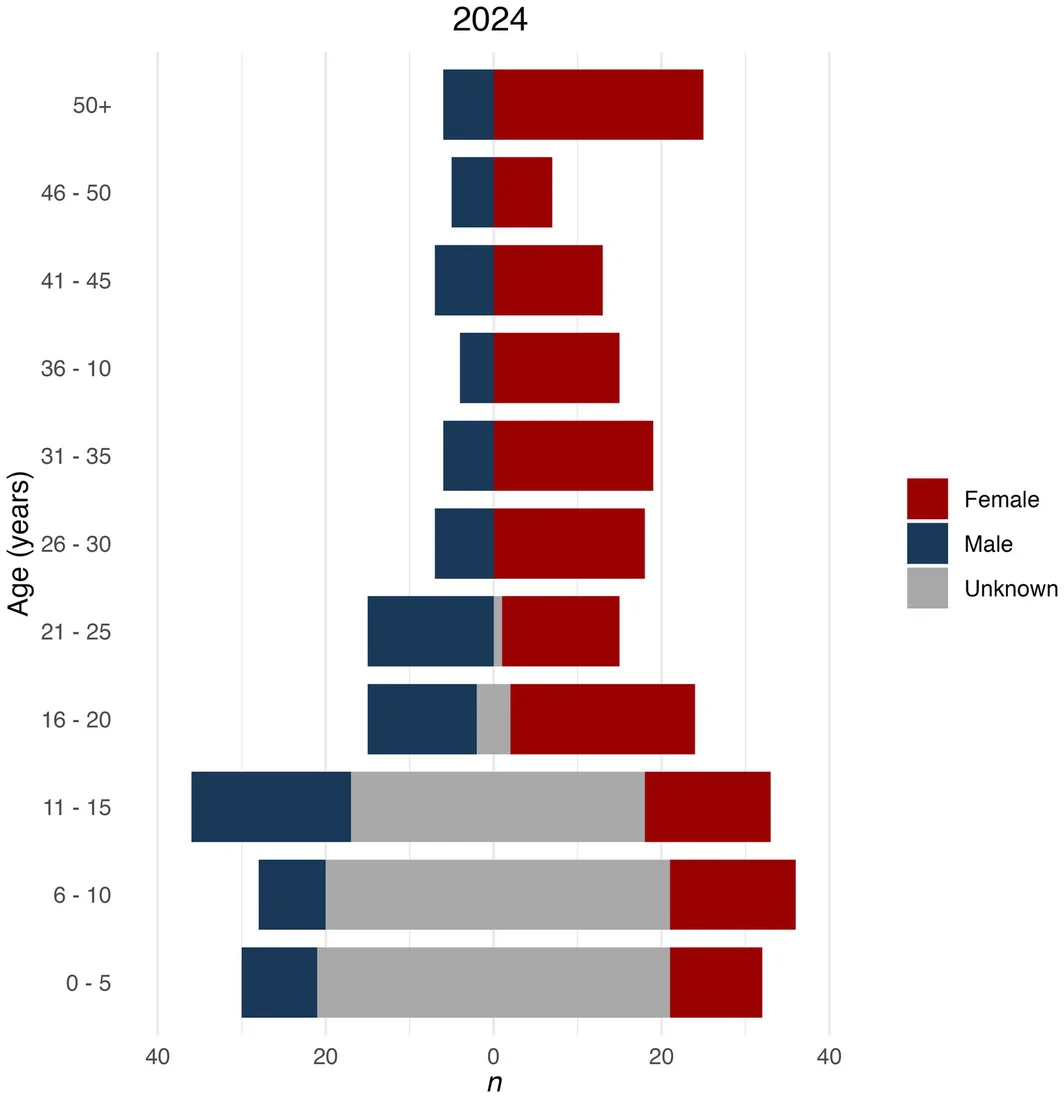
Age and sex structure of 396 presumed living, photo‐identified West Coast Transient (WCT) killer whales at the end of 2024. Sex was often unknown prior to the onset of maturation but was applied retroactively when confirmed. Individuals of unknown sex (gray) were equally allocated across male (blue) and female (red) axes.

WCT were photographed in 9427 encounters between 1972 and 2024. Encounters occurred year‐round but were most common in summer months; over 60% of all encounters analyzed occurred between June and September. Encounters were photographed by researchers at Fisheries and Oceans Canada, Bay Cetology, and many partner organizations, as well as by contributing members of the public (list of contributors available in Towers et al. 2019, 2025). Survey effort and community contributions increased over the study period from an average of 7.3 encounters per year in the first 10 years of the study (1972–1981) to over 950 encounters in 2024 alone. These encounters resulted in a total of 580 individually‐identified WCT (Table S1), 396 of which were assumed to be alive at the end of 2024 (385 represented in the 2024 coastal subset): 174 females, 99 males, and 123 individuals of unknown sex, most of whom were juveniles under 15 years of age (Figure 2; Towers et al. 2019, 2025).

### 3.2 Recruitment

**FIGURE 3:**
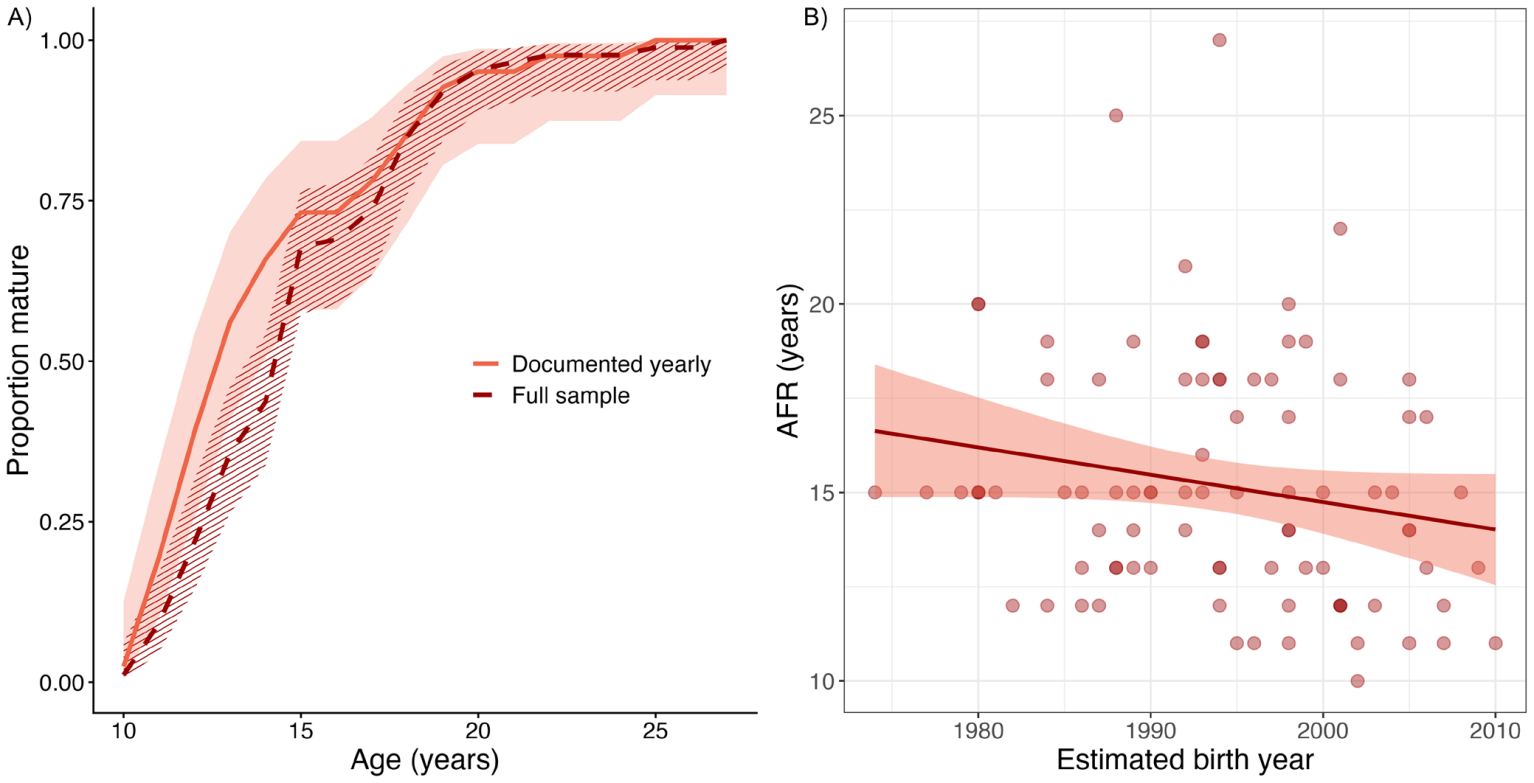
(A) Maturity‐at‐age proportions for known‐age female West Coast Transients (WCT; *n* = 87, dashed line) and a subsample with no annual observational gaps (*n* = 41, solid line). Shaded/hatched regions indicate 95% confidence intervals estimated using the Wilson method in the R package *binom* (Brown et al. 2001; Dorai‐Raj 2022). (B) Age of first recruitment (AFR) for 87 known‐age female WCT killer whales with respect to estimated birth year. Points are semitransparent to show overlap (i.e., darker points indicate more observations). Predictions (line) and 95% confidence interval (shaded region) from a marginally significant linear model (*AFR* ~ *BY*; regression slope = −0.07, *p* = 0.08) are overlaid.

A total of 221 female WCT were identified, of which 87 gave birth and recruited a viable first calf between 1972 and 2023 (Table S2). Seven females in this sample gave birth to one or more nonviable calves prior to recruiting a viable calf. Age of first recruitment was seen as early as age 10 and as late as age 27 (Figure 3A). An 8‐year‐old female, T075B2, was documented with a new calf in 2023, but within a month of its detection, the calf was photographed in a state of emaciation, after which it disappeared and was pronounced dead. This birth suggests that WCT females may be sexually mature as early as ~7 years. Mean age of first recruitment of WCT was estimated at 15.2 years (bootstrap SE = 0.4 years, 95% CI = 14.5–15.9 years; Table 1). We also estimated age of first reproduction in WCT using 97 females that were born and gave birth to any first calf (viable or nonviable) between 1972 and 2024 (Table S2). Mean age of first reproduction in WCT was estimated at 15.1 years (SE = 0.4 years) with a range of 8 to 28 years.

**TABLE 1:** Mean (±SE) age of first recruitment (AFR, in years) and age of first reproduction (AFRP, in years) for three populations of killer whales in the eastern North Pacific: West Coast Transient killer whales (WCT, 1972–2024; this study), Northern Resident killer whales (NRKW) in a period of increasing abundance (1973–1996; Olesiuk et al. 2005) and a period of no net population growth (1996–2004; Olesiuk et al. 2005), and Southern Alaska Resident killer whales (SARKW, 1984–2010; Matkin et al. 2014).

Population	AFR	AFRP
WCT	15.2 (0.4)	15.1 (0.2)
NRKW^a^ (increasing abundance)	14.1 (0.2)	NA
NRKW^a^ (no net growth)	15.4 (0.3)	NA
SARKW^b^	NA	12.8 (0.2)

^a^ Olesiuk et al. (2005).

^b^ Matkin et al. (2014).

The mean observational gap in our WCT sample was 2.8 years (median 2 years, range 0–13 years). To account for the possibility of undocumented calves that could artificially inflate estimates of AFR, we also estimated mean AFR for a subsample of 41 females with no yearly observational gaps in the reproductive years leading up to the birth of their first viable calf (i.e., a minimum of one documentation per year from age 10 up to first recruitment; Table S2). The estimated difference in mean AFR between the subsample and the full sample was statistically nonsignificant (bootstrap with replacement, 1000 resamples, 95% CI of difference in means = −0.41–2.13; Figure 3A). We also found a marginally significant effect of birth year (*BY*; range 1974–2010) on AFR (*AFR* ~ *BY*; regression slope = −0.07, *p* = 0.08), indicating a small decline in the age at which female WCT matured over the course of this study (Figure 3B), though this could have been biased by increasing encounter frequency in the later years of the survey.

### 3.3 Fecundity

**FIGURE 4:**
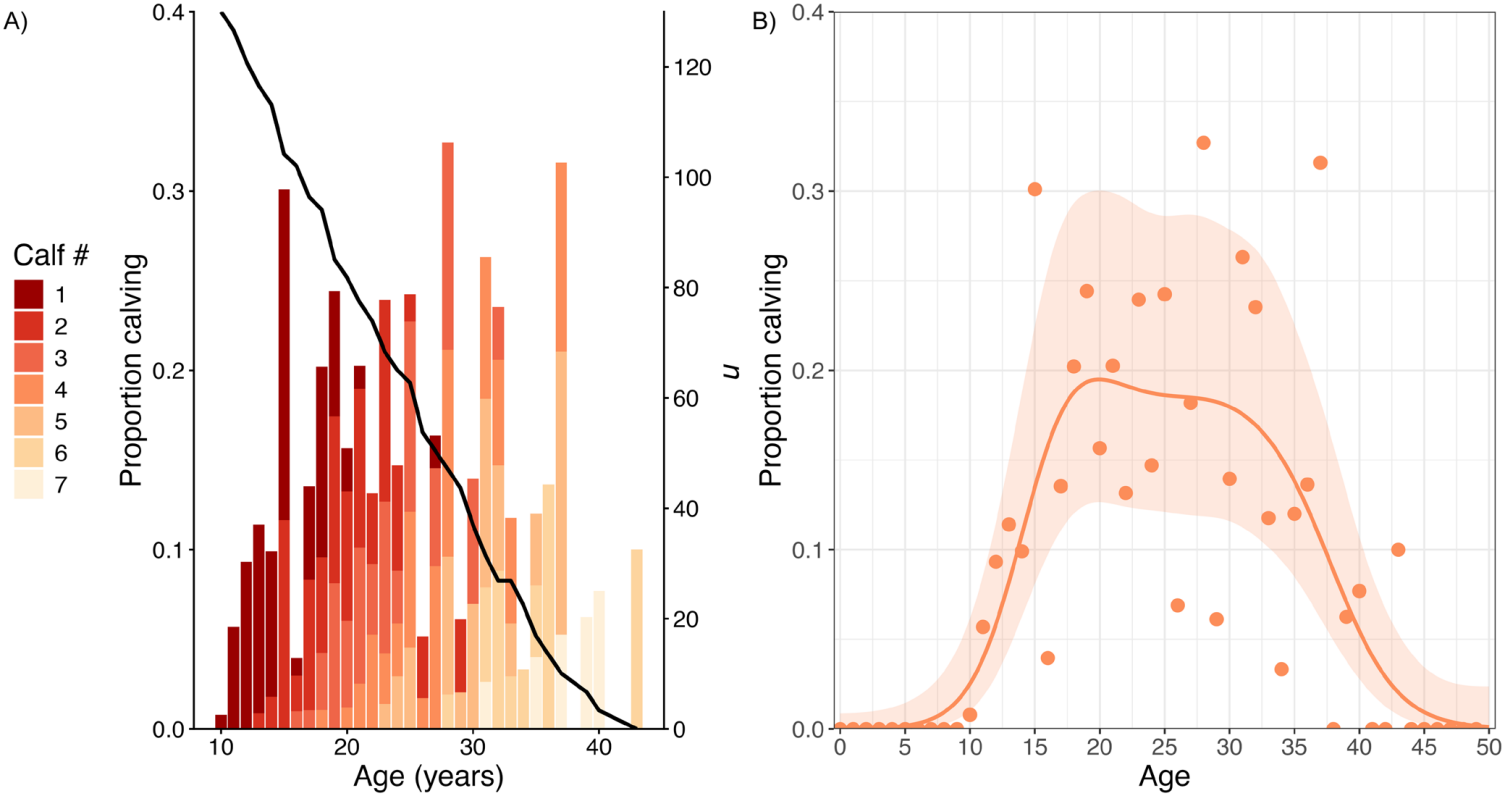
(A) Proportions of female West Coast Transient (WCT) killer whales of a given age that had a viable (surviving ≥ 1 year) calf at that age (columns; left y‐axis). Stacked columns indicate proportions with respect to order of calves (inclusive of all offspring, including those that did not survive to year 1). Sample sizes are overlaid in black (right y‐axis). (B) Predicted age‐specific fecundity (*FEC*_*f*(*x*)_) of female WCT (*FEC*_*f*(*x*)_ ~ *age*) with 95% confidence interval (shaded region). Actual proportions of female WCT killer whales of a given age that had a viable (surviving ≥ 1 year) calf at that age are shown as points.

Age‐specific fecundity (*FEC*_*f*(*x*)_) was estimated for 153 female WCT that were born during the survey period or < 10 years old at its beginning, representing a total of 3306 animal‐years analyzed. Fecundity was examined from ages 0 to 50, though viable calves were only born to females between the ages of 10 and 43 and sample size declined with age (maximum *n* = 126 at age 8; Figure 4A). When pooled into decadal age classes, fecundity of WCT females declined after ages 20–29. When pooled into age classes defined by Vélez‐Espino et al. (2014), mean fecundity of WCT was 0.14 (SE = 0.01) from ages 10–29 and 0.13 (SE = 0.02) from ages 30–50. The GAM smooth term for age had *edf* = 4.91 (*p* < 0.0001) and explained 70% of residual deviance (Figure 4B; adjusted *R* ^2^ = 0.55).

**TABLE 2:** Mean fecundity (±SE) of female West Coast Transient killer whales (WCT; this study) and female Northern Resident killer whales (NRKW; Olesiuk et al. 2005) in a period of unrestricted population growth (1973–1996) and a period of population fluctuation with no net growth (1996–2004). Standard errors were not reported for NRKW.

Age class (years)	WCT	NRKW (1973–1996)^a^	NRKW (1996–2004)^a^
10–19	0.12 (0.01)	NA	NA
20–29	0.18 (0.01)	0.16	0.15
30–39	0.15 (0.02)	0.12	0.13
40–49	0.03 (0.02)	0.05	0.05

^a^ Olesiuk et al. (2005).

We documented 256 calving intervals among 385 viable WCT calves born to 98 different females between 1982 and 2023 (Table S3). Calving intervals ranged from 2 to 16 years (median 5 years), and the mean calving interval was estimated at 4.88 years (SE = 0.13 years) resulting in an average annual calving rate of 0.20 calves per reproductive female per year. We identified a total of 128 potentially reproductive females (i.e., alive and greater than 10 years old) during the study period, but as of the end of 2024, 22 had not yet been observed with any calves, viable or nonviable. Five females in this sample only gave birth to nonviable calves.

**FIGURE 5:**
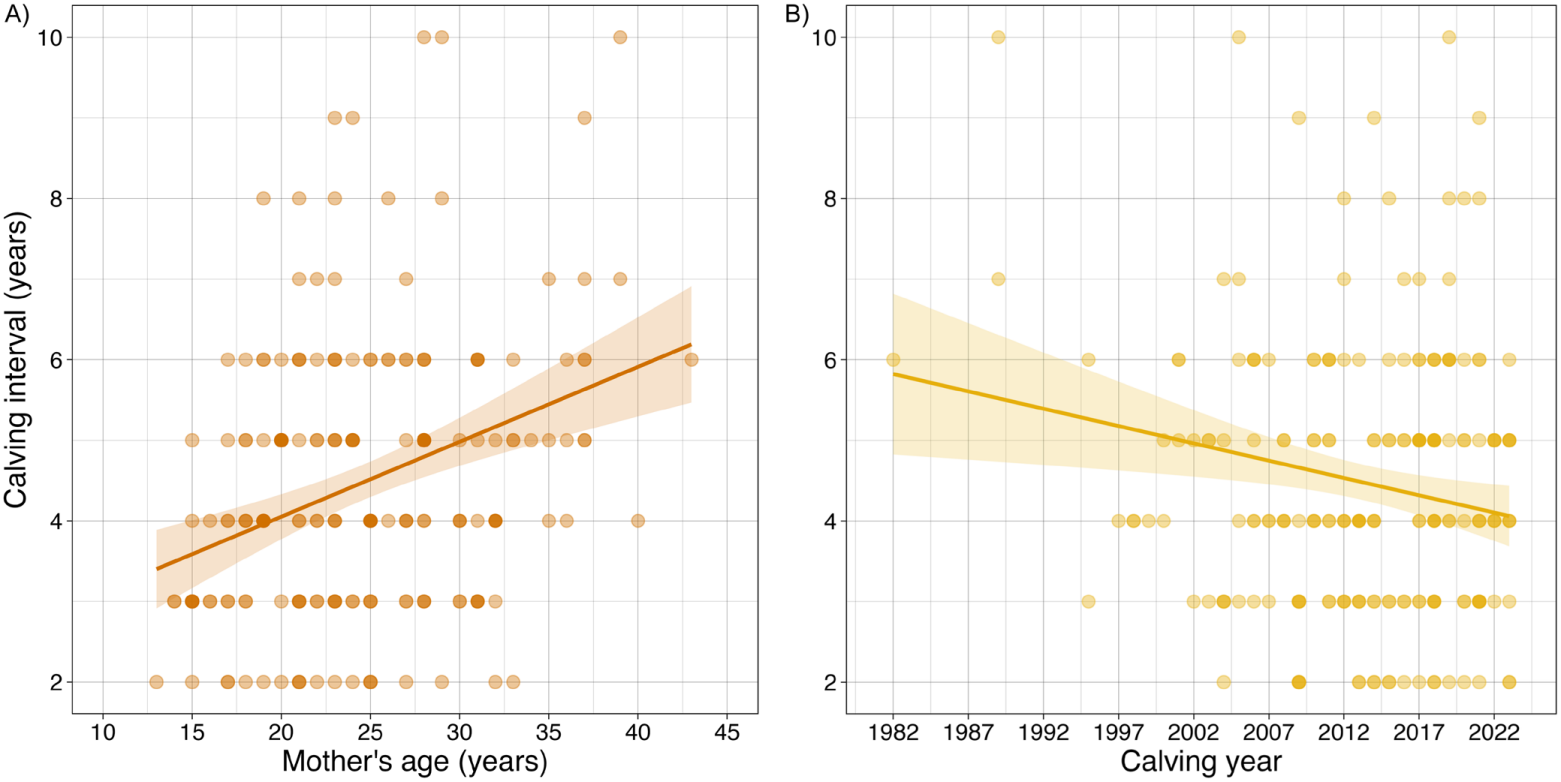
(A) West Coast Transient (WCT) killer whale calving interval duration with respect to maternal age and (B) calving year for 256 calving intervals among 385 viable calves. Points are semitransparent to show overlap (i.e., darker points indicate more observations). Lines with shaded regions represent predictions and 95% confidence intervals from the AICc‐best multiple linear regression model of calving interval length (*CI* ~ *MAB* + *CY*; Table S4).

There were significant effects of maternal age at birth and calving year on calving interval length. The AICc‐best and simplest model regressed calving interval length on maternal age at birth and calving year (Figure 5), though ΔAICc among the six best candidate models was < 2 units (Table S4). In the selected model, WCT calving intervals increased with increasing maternal age (regression slope = 0.093, *p* < 0.0001; Figure 5A). Mean WCT calving interval decreased over the study period (regression slope = −0.04, *p* = 0.008) from a mean of 6.78 years (SE = 1.02 years) in the 1980s to 4.66 years (SE = 0.17 years) in the 2010s (Figure 5B). Yearly encounter rates increased over the study period and could have affected these estimates by impacting probability of detection, but our sensitivity analyses indicated that both effects were robust to changes over time: in 1000 iterations of randomly resampling up to 3 calving intervals per calving year and fitting our best candidate model, maternal age at birth was a significant predictor of calving interval length in 99.5% of simulations, while calving year was a significant predictor of calving interval length in 88.3% of simulations.

We also estimated mean calving interval for a subsample of 100 calving intervals among 162 viable calves that were born from mothers documented at least every other year (Table S3). These calves were born between 1995 and 2023 from 35 different females. Calving intervals in this subsample ranged from 2 to 10 years (median 4 years) and the mean calving interval was estimated at 4.13 years (SE = 0.17 years), resulting in an average annual fecundity rate of 0.24 calves per female per year. There was evidence of difference between this estimate and the average annual calving interval estimated for the entire sample (bootstrap with replacement, 1000 resamples, 95% CI of difference in means = 0.34–1.16), though the difference likely reflects the shorter, more recent time interval represented by the subsample.

### 3.4 Mortality

**FIGURE 6:**
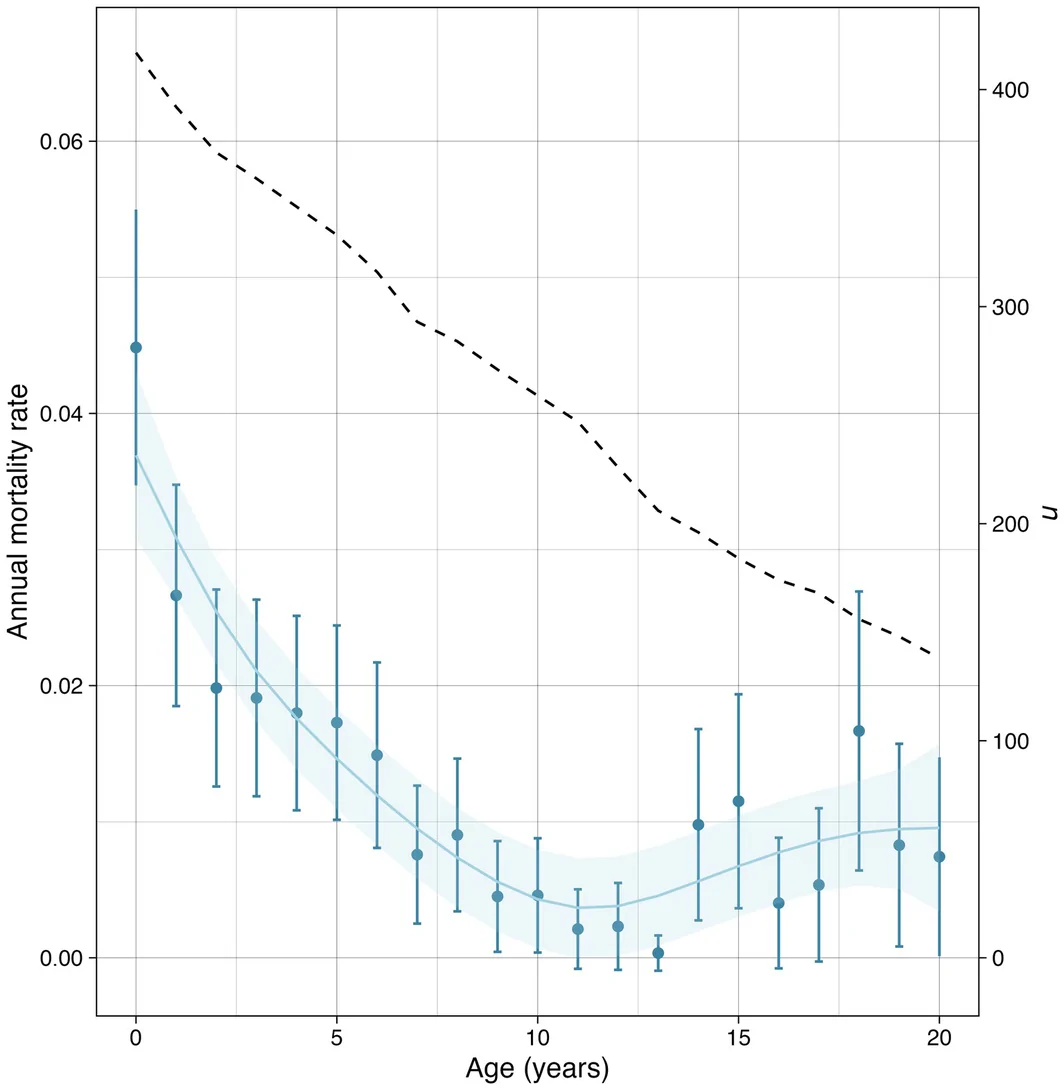
Estimated annual mortality rate (AMR) of 464 West Coast Transient (WCT) killer whales born 1972–2024 (dark blue points, left y‐axis), with standard error bars (dark blue lines, left y‐axis) and sample sizes for each age (*n*, black dashed line, right y‐axis). Predicted AMR (light blue solid line, left y‐axis) and 95% confidence interval (shaded region, left y‐axis) from a GAM smooth overlaid (*AMR* ~ *age*).

Annual mortality rates from ages 0–20 were calculated using 464 WCT born during the study period (Figure 6). Status (living/dead) was evaluated across 4230 animal‐years. A GAM smooth was fit to annual mortality rate using thin‐plate regression splines, a Gaussian error distribution, and restricted maximum likelihood via *mgcv* (*AMR* ~ *age*; Figure 6; Wood 2017). The smooth term for age had *edf* = 4.01 (*p* < 0.0001) and explained 86% of residual deviance (adjusted *R* ^2^ = 0.83). Annual mortality rate was also calculated for each of the progressively widening age classes used by Murray et al. (2021) and Vélez‐Espino et al. (2014). Annual mortality was greatest in the youngest age class (Table 3, Figure 6).

## 4 Discussion

This study integrated more than 50 years of photo‐identification encounters with WCT to assess life history parameters over multiple generations of whales. In combination with previously published estimates for sympatric Resident killer whale populations, our estimates may be particularly useful for examining the ecological significance of life history in a long‐lived species complex. While many aspects of life history were apparently conserved across killer whale ecotypes, some subtle differences in WCT estimates aligned with long‐term observations of increasing abundance in a coastal subset of this population. These key differences may reflect divergent evolution among discrete ecotypes or variations in survey effort within or among studies, but could also indicate population‐specific vulnerabilities in overlapping habitat, including discrepancies in prey preference and availability, contamination levels, social behavior, and exposure to anthropogenic impacts.

### 4.1 Comparison With Sympatric Populations

Reproductive parameter estimates for WCT were mostly within the range of Resident killer whale populations. However, we found small, key differences in certain WCT parameters that describe some of the demographic mechanisms of increasing abundance. Mean age of first recruitment in WCT was consistent with NRKW during a period of no net population growth, but slightly exceeded the estimate for NRKW during an earlier period of population increase (Table 1; Olesiuk et al. 2005). Similarly, mean age of first reproduction in WCT exceeded the estimate reported for SARKW, indicating that WCT may begin calving slightly later than SARKW (Table 1, Matkin et al. 2014). However, when we regressed age of first recruitment on birth year, we found marginal evidence of a small decrease in the ages at which female WCT recruited their first calf over the course of this study (*AFR* ~ *BY*; regression slope = −0.07, *p* = 0.08), suggesting that recruitment may be occurring earlier in WCT in recent years. By contrast, mean age of first recruitment of NRKW increased between a period of unrestrained population growth (1973–1996) and a period of no net population change (1996–2004; Olesiuk et al. 2005). While subtle, such changes in age of first recruitment have important bearing on population dynamics by impacting the duration of female reproductive activity. Even a small decline in the age at which WCT recruit their first calf could result in a biologically significant increase in calf‐bearing years at the population level, supporting a trend of increasing abundance.

Fecundity of younger female WCT was similar to or greater than sympatric Resident populations, but declined with age, dropping to 0 between the ages of 45 and 50. This decline has also been noted in Resident killer whales and is consistent with the onset of reproductive senescence (Table 2; Matkin et al. 2014; Olesiuk et al. 2005; Nielsen et al. 2021). WCT in their 20s and 30s had slightly greater mean fecundity than NRKW, while WCT in their 40s had lower mean fecundity than NRKW (Table 2; Olesiuk et al. 2005). According to bins defined by Vélez‐Espino et al. (2014), “young” reproductive WCT females (aged 10–29) were more fecund than SRKW, while fecundity of “old” WCT females (aged 30–50) exceeded that of SRKW and NRKW (Vélez‐Espino et al. 2014). However, our sample size for WCT in their 40s and older was small, as we restricted analyses of age‐specific fecundity to females that matured during the study period. WCT fecundity was broadly in range of or higher than that of Residents, as was anticipated for a growing population, but further observations of older known‐age WCT would be needed to draw strong conclusions about late‐stage fecundity in this population.

Calving interval lengths were also broadly indicative of overall population dynamics across WCT and Residents: mean calving interval for WCT was similar to SARKW and to NRKW during their period of population growth, but was shorter than SRKW and NRKW during their period of no net population change (Krahn et al. 2002; Matkin et al. 2014; Olesiuk et al. 2005). Variability in calving intervals was also lower in WCT than in other populations, though the ranges (2–16 years in WCT, 2–14 years in SARKW, 2–14 years in NRKW) were comparable. Aligning with our observations of decreasing fecundity with age, we found evidence of a small increase in WCT calving interval length with increasing maternal age (regression slope = 0.093, *p* < 0.0001; Figure 5A). A similar pattern has been shown for SARKW (Matkin et al. 2014) and for bottlenose dolphins ( *Tursiops truncatus* ) in Sarasota Bay (Wells et al. 2025), though Olesiuk et al. (2005) found no evidence of variation in calving interval length with maternal age in NRKW. We also found evidence that mean WCT calving interval decreased slightly over the study period (regression slope = −0.04, *p* = 0.008; Figure 5B). By contrast, Matkin et al. (2014) and Olesiuk et al. (2005) did not find evidence for changes in calving interval length for SARKW or NRKW during their respective study periods. This trend may have important bearing on the WCT population over time: shorter calving intervals can result in more offspring per female over the course of her reproductive lifetime, bolstering recruitment and abundance.

It is important to note that decreasing age of first recruitment, high fecundity, and shortening calving intervals are all innately linked to WCT calf survivorship. While age‐specific mortality of WCT follows a typical bathtub‐shaped mammalian trajectory (Figure 6; Nielsen et al. 2021) and was highest in the first years of life for all populations assessed, WCT had the lowest rate of infant mortality (Table 3). This comparatively strong rate of WCT survival to age 1 directly affected our estimates of fecundity and recruitment via the consideration of calf viability. Consequently, close examination of the underlying ecological and evolutionary conditions supporting calf survival, as well as the other notable aspects of WCT life history described above, can shed light on population‐specific underpinnings of increasing abundance.

**TABLE 3:** Mean (±SD) annual mortality rates for four populations of killer whales in the eastern North Pacific: West Coast Transient killer whales (WCT, 1972–2024; this study), Southern Resident killer whales (SRKW, 1987–2011; Murray et al. 2021; Vélez‐Espino et al. 2014), Northern Resident killer whales (NRKW, 1987–2011; Murray et al. 2021; Vélez‐Espino et al. 2014), and Southern Alaska Resident killer whales (SARKW, 1984–2010; Matkin et al. 2014). We represented standard deviations rather than standard errors due to unpublished sample sizes for SRKW and NRKW.

Age class (years)^a^	WCT	SRKW^1^, ^2^	NRKW^1^, ^2^	SARKW^3^
0–1	0.045 (0.009)	0.215 (0.284)	0.078 (0.082)	0.054 (0.244)
1–2	0.026 (0.007)	0.019 (0.047)	0.028 (0.019)	0.003 (0.040)
2–5	0.019 (0.003)	0.019 (0.047)	0.028 (0.019)	0.010 (0.054)
5–10	0.011 (0.002)	0.019 (0.047)	0.028 (0.019)	0.012 (0.064)
10–16	0.005 (0.001)	0.015 (0.033)	0.011 (0.012)	0.008 (0.032)

^1^ Vélez‐Espino et al. (2014).

^2^ Murray et al. (2021).

^3^ Matkin et al. (2014).

^a^ In Matkin et al. (2014), age classes were defined between half‐years (e.g., from 0.5 to 1.5 years) and the eldest juvenile age‐class was defined from 10.5 to 14.5 years. In Murray et al. (2021) and Vélez‐Espino et al. (2014), age classes were defined between integer years (e.g., age 0–1) and the eldest juvenile age‐class was defined from 10 to 16 years.

### 4.2 Evolutionary and Ecological Context

Consistency in certain parameters and patterns of life history (age of maturation, mean calving interval, declining fecundity with age) indicates that many aspects of killer whale ecology are basal and conserved across populations and ecotypes. Small differences in other aspects of life history, including temporal shifts, may therefore reflect subtle responses to environmental pressures that affect ecotypes differently as a result of their divergence (Esteban et al. 2025). In particular, ecotype‐specific dietary preferences result in major differences in prey availability and quality between WCT and Resident killer whale populations. Salmon specialist SRKW have experienced declines in prey availability in the last several decades, which have been linked to poor recruitment, low abundance, and reduced body condition (Ford et al. 2009; Hanson et al. 2021; Stewart et al. 2021; Wasser et al. 2017). In the same time frame, abundances of several important WCT prey species have increased within their range and have been linked to robust WCT growth and body condition (Elliser and Hall 2021; Jefferson et al. 2023; Kotik et al. 2022; Laake et al. 2018; Carretta et al. 2023; Majewski et al. 2024; Pearson et al. 2025). Strong prey availability and excellent body condition are critical to successful recruitment, particularly for females bearing calves in close succession, e.g., within 2–3 years, as was observed for over 27% of WCT calving intervals. Accounting for a 15–18‐month gestation and 1–2 years of nursing, 2–3‐year calving intervals signify simultaneous gestation and lactation in killer whales, a particularly impressive feat of energetic performance requiring a substantial boost in caloric intake (Kastelein et al. 2003; Kriete 1995). Improved prey abundance likely also contributes to increased calf survivorship, as calf loss in killer whales can and does occur when females are unable to support themselves and a growing infant. Nutritional stress from low prey availability has been linked to high rates of abortion and low calf survivorship in SRKW, whose infant mortality rates were more than double those of any other population in this study (Ward et al. 2009; Wasser et al. 2017). Comparatively high rates of infant survival in WCT align with observed population dynamics and likely contributed to the nearly 32% reduction in WCT mean calving interval duration from the 1980s to the 2010s. Similarly, it is unlikely that changes in WCT age of first recruitment represent changes in growth or reproductive physiology: in both WCT and NRKW, age of first recruitment was consistent with the slowdown of somatic growth, which generally occurs from ages 14–15 (Fearnbach et al. 2011; Kotik et al. 2022). Instead, the decline in age of first recruitment in WCT may have resulted in part from improved survival of firstborn calves in recent years. A variety of other environmental conditions, such as decreasing concentrations of toxic anthropogenic pollutants known to transfer from female whales to calves, could have affected infant mortality and related life history parameters (Andvik et al. 2021; Lee et al. 2022; Ross et al. 2013; Ylitalo et al. 2001), but further study is required to tease out specific effects of contaminants, prey availability, and other environmental cofactors on killer whale fitness and life history outcomes.

Differences in life history parameters among killer whale populations may also be driven in part by behavioral divergence between ecotypes. Killer whales are not generally thought to experience calf loss from predation, but infant mortality may occur from intraspecific calf‐directed aggression. Infanticide has been documented in the WCT population (Towers et al. 2018); postpartum interactions and scar acquisition rates also suggest that multiple killer whale populations may exhibit calf‐directed aggression (Grimes et al. 2022; Stacey and Baird 1997; van Weelden et al. 2025). Infanticide is poorly observed and not currently known to significantly impact calf survivorship, but differential rates of calf‐directed aggression, possibly facilitated or prevented by differences in social structure, could affect comparative rates of juvenile survivorship. Unlike Resident killer whales, WCT often disperse from their maternal kin, a behavior thought to help maintain smaller, more efficient hunting parties (Baird and Whitehead 2000; Nielsen et al. 2023). Differences in group size could exacerbate or ameliorate the effects of calf‐directed aggression, and WCT calves may also benefit from lessened competition for resources in comparatively smaller family groupings than are generally experienced by Resident killer whale calves. While difficult to quantify, differences in social behavior and their effects on life history, particularly survival, should not be discounted when comparing between ecotypes.

### 4.3 Study Limitations

We estimated WCT life history parameters using uniquely valuable data derived from one of the longest‐running cetacean monitoring efforts known, but our sample sizes were restricted and these data were not without limitation. As is often the case for long‐running studies with data sourced from many different contributors, survey effort was inconsistent, but the data required to effectively incorporate effort as a model covariate (e.g., completeness of photographic coverage across WCT encounters, survey hours, and spatial coverage) were not available for the majority of encounters. Furthermore, we did not have access to data on survey effort in sympatric populations, restricting our ability to determine how such variation may have influenced points of comparison. As a result, these analyses should be interpreted with some caution, as differences in detection probability could have biased the results. For example, comparatively low encounter frequencies in the 1970s, 1980s, and 1990s could have affected our ability to detect changes in calving interval length and age of first recruitment over the study period. Differences in effort and resultant detection bias can also affect estimates of mortality rates in killer whale calves, though these are likely underestimated across populations, as not all calves are detected. Our life history estimates were also calculated at a temporal resolution of one calendar year that may have masked seasonal effects. Births have rarely been directly observed, but there is evidence that Resident killer whale reproductivity in the eastern North Pacific may be at least somewhat seasonal (Stacey and Baird 1997; Olesiuk et al. 2005) and it is possible that WCT also calve on a seasonal basis. Our comparisons with Resident killer whale populations may have been impacted by uncertain age estimates in previously‐published analyses of life history (Bateman et al. 2025). Additionally, age of first recruitment, as defined by the birth of the first viable calf, could have been inflated by calves that did not survive long enough to be detected. We did not find evidence of effects from gaps in observational records on age of first recruitment and calving intervals, but increased frequency of detection may not have entirely accounted for this hidden bias, which could have led to overestimation of calving intervals. It should also be noted that the WCT individuals used to estimate these life history parameters (Table S1) do not represent the entire WCT population, the size of which is not currently known.

We also did not attempt to estimate the age of maturation of male WCT in this study. Olesiuk et al. (2005) noted that the growth or “sprouting” of the dorsal fin of a male killer whale occurs as a secondary sexual characteristic symptomatic of increased testosterone production at the onset of puberty. Accordingly, maturation of male killer whales has previously been defined as the age at which the dorsal fin attains a height to width ratio exceeding 1.4, at which point it is easily distinguished from the fin of a mature female. However, physical maturation may not necessarily indicate actual reproductive success in male killer whales, which is theorized to increase with increasing age in SRKW (Ford et al. 2018) but is not well understood in other populations and ecotypes. Ultimately, little is known about the exact timing of sexual maturation of male WCT and we did not attempt to characterize it. We were also unable to partition juvenile survivorship by sex, as killer whale sexes are often not identified until maturity. However, sex‐specific survivorship trends should not be discounted, particularly given the established literature on interactions between sociality and survivorship in this species complex (Foster et al. 2012; Nattrass et al. 2019; Nielsen et al. 2023; van Weelden et al. 2025). Continued monitoring, along with genetic identification of sex from biopsied tissues, may enable expanded analyses going forward.

### 4.4 Conclusions

We found that WCT shared many aspects of life history with Resident killer whales despite the ecological and genetic differences between the two ecotypes (Baird and Whitehead 2000; Bigg 1982; Bigg et al. 1990; Ford and Ellis 1999; Morin et al. 2024). Best estimates place the initial divergence of Bigg's and Resident killer whales approximately 350,000 years ago (Morin et al. 2015), during which time the two lineages have developed unique feeding, social, and acoustic behaviors, as well as differences in morphology that are sufficient for experienced observers to visually identify whales to ecotype (Baird and Dill 1995; Bigg 1982; Emmons et al. 2019; Ford et al. 1998; Morton 1990). Amid these differences, consistency in certain aspects of life history (age of maturation, mean calving interval, declining mortality with age) indicates that many aspects of killer whale ecology are basal and conserved. Small differences in other aspects of life history (calving interval dynamics, early life mortality) likely reflect subtle responses to environmental pressures that affect these ecotypes differently as a result of their behavioral divergence, particularly with regard to prey preference.

Increasing abundances of WCT in the coastal waters of the eastern North Pacific have prompted inquiries into when this population may approach carrying capacity (Murray et al. 2025; Towers et al. 2025). However, the extended lifespan, low recruitment rate, and uniparity of killer whales at the species level makes them slow to respond to subtle perturbations in resource availability or to show evidence of density dependence (Matkin et al. 2014; Olesiuk et al. 2005). Instead, a protracted period of population‐level response may be observed as prey availability and other environmental conditions impact the survival and recruitment of WCT in this region. Continued comparisons between WCT and overlapping killer whale ecotypes, as well as ongoing monitoring through photo‐identification with increasing contributions from community scientists via finwave.io, will be invaluable for future analyses of these and other killer whale populations.

## Author Contributions

**Chloe Kotik:** conceptualization (lead), data curation (supporting), formal analysis (lead), funding acquisition (equal), visualization (lead), writing – original draft (lead), writing – review and editing (lead). **Thomas Doniol‐Valcroze:** conceptualization (equal), formal analysis (supporting), funding acquisition (equal), project administration (supporting), supervision (equal), writing – review and editing (equal). **Jared R. Towers:** conceptualization (equal), data curation (lead), project administration (lead), supervision (equal), writing – review and editing (equal). **Lara Horstmann:** funding acquisition (equal), supervision (lead), writing – review and editing (equal), writing – review and editing (equal).

## Funding

This work was supported by North Pacific Research Board, University of Alaska Fairbanks, Fisheries and Oceans Canada and Orca Conservancy.

## Conflicts of Interest

The authors declare no conflicts of interest.

## Supporting information


**Table S1:** IDs of 580 West Coast Transient (WCT) killer whales used to estimate life history parameters.
**Table S2:** ID and estimated birth years for 97 female West Coast Transient (WCT) killer whales used to estimate age of first recruitment (AFR, in years, inclusive of viable calves only) and age of first reproduction (AFRP, in years, inclusive of all calves). WCT that gave birth to a first calf in 2024 were not included in estimation of AFR, as a full year had not passed to determine whether those calves should be considered viable. A subsample of 41 females (indicated with *), which were documented at least once per year between age 10 and first recruitment, was used to calculate AFR separately and test for evidence of difference.
**Table S3:** ID and estimated birth year for 98 female West Coast Transient (WCT) killer whales used to estimate mean calving interval and average annual fecundity rate. Female WCT that were born prior to the beginning of the survey (or that were physically mature at first documentation) were assigned a maximum birth year ≥ 10 years prior to the year in which they were first observed (Towers et al. 2019). We also separately estimated mean calving interval and annual fecundity rate from a subsample of calving intervals from 35 female WCT that were documented at least every other year beginning from age 10 (indicated with *).
**Table S4:** Model selection criteria for the six best candidate models examining effects of West Coast Transient (WCT) maternal age and year of calving on calving interval (CI) length. AICc: small‐sample Aikaike's Information Criterion. Only models with ΔAICc < 2 units are shown.

## Data Availability

Data to perform life history analyses on West Coast Transient (WCT) killer whales may be found in Appendix Tables S1–S3 or at www.finwave.io.
