# Supplementary material for: Life History and Reproductive Parameters in an Increasingly Abundant Killer Whale Population
Source: Ecol Evol. 2026 Aug 19;16(8):e74185. doi: 10.1002/ece3.74185 (PMC13489756; doi:10.1002/ece3.74185)
Supplement: Supplementary file 1 [file ECE3-16-e74185-s001.docx]

**Appendix**

**Table S1:** IDs of 580 West Coast Transient (WCT) killer whales used to estimate life history parameters.

| **ID** | | | | |
| --- | --- | --- | --- | --- |
| T001 | T036B1 | T061 | T084 | T124A3A |
| T002 | T036B2 | T062 | T085 | T124A4 |
| T002A | T036B3 | T063 | T085A | T124A4A |
| T002B | T036B4 | T064 | T085B | T124A5 |
| T002B1 | T036C | T064A | T085C | T124A6 |
| T002B2 | T037 | T064A1 | T085D | T124A7 |
| T002C | T037A | T064A2 | T086 | T124B |
| T002C1 | T037A1 | T064A3 | T086A | T124B1 |
| T002C2 | T037A2 | T064A4 | T086A1 | T124C |
| T002C3 | T037A3 | T064A5 | T086A1A | T124D |
| T002C4 | T037A4 | T064B | T086A1B | T124D1 |
| T002C5 | T037A5 | T064B1 | T086A2 | T124D2 |
| T006 | T037B | T064B1A | T086A3 | T124D3 |
| T007 | T037B1 | T064B2 | T086A3A | T124D4 |
| T007A | T037B2 | T064C | T086A4 | T124E |
| T007B | T037B3 | T065 | T086A5 | T125 |
| T007B1 | T037C | T065A | T087 | T125A |
| T007B2 | T038 | T065A1 | T088 | T126 |
| T007B3 | T038A | T065A2 | T089 | T127 |
| T007B4 | T038A1 | T065A3 | T090 | T128 |
| T007B5 | T038A2 | T065A4 | T090A | T132 |
| T007B6 | T038A3 | T065A5 | T090B | T134 |
| T007B7 | T038B | T065A6 | T090C | T136 |
| T007C | T038B1 | T065B | T090D | T137 |
| T010 | T038B2 | T065B1 | T091 | T137A |
| T010A | T038C | T065B2 | T092 | T137B |
| T10B | T038D | T065B3 | T093 | T137C |
| T010C | T038E | T066 | T094 | T137D |
| T011 | T038F | T067 | T095 | T138 |
| T011A | T039 | T068 | T096 | T139 |
| T012 | T040 | T068A | T097 | T140 |
| T012A | T041 | T068B | T098 | T140A |
| T012B | T041A | T068B1 | T099 | T140B |
| T012C | T041A1 | T068B1A | T099A | T140C |
| T013 | T041A2 | T068B1B | T099B | T141 |
| T014 | T041A3 | T068B1C | T099B1 | T141A |
| T015 | T042 | T068B2 | T099C | T141B |
| T016 | T044 | T068B3 | T099D | T141C |
| T017 | T045 | T068B4 | T099E | T141D |
| T018 | T046 | T068B5 | T100 | T142 |
| T019 | T046A | T068C | T100A | T143 |
| T019A | T046B | T068C1 | T100B | T144 |
| T019B | T046B1 | T068C1A | T100B1 | T145 |
| T019C | T046B1A | T068C2 | T100B2 | T146 |
| T020 | T046B1B | T068C3 | T100B3 | T146A |
| T021 | T046B1C | T068C3A | T100C | T146A1 |
| T021A | T046B2 | T068C4 | T100D | T146A2 |
| T022 | T046B2A | T068C5 | T100E | T146B |
| T022A | T046B2B | T068C6 | T100F | T146C |
| T023 | T046B3 | T068D | T101 | T146C1 |
| T023A | T046B4 | T069 | T101A | T146D |
| T023B | T046B5 | T069A | T101B | T146E |
| T023C | T046B6 | T069A1 | T102 | T147 |
| T023C1 | T046B7 | T069A2 | T103 | T148 |
| T023C2 | T046C | T069A3 | T104 | T149 |
| T023C3 | T046C1 | T069A4 | T105 | T150 |
| T023C4 | T046C2 | T069A5 | T107 | T151 |
| T023C5 | T046C3 | T069A6 | T108 | T151A |
| T023C6 | T046C4 | T069B | T109 | T151A1 |
| T023D | T046D | T069C | T109A | T151B |
| T023D1 | T046E | T069D | T109A1 | T151C |
| T023D2 | T046F | T069D1 | T109A2 | T151D |
| T023D3 | T047 | T069D2 | T109A2A | T152 |
| T023D4 | T048 | T069D3 | T109A2B | T152A |
| T023D5 | T049 | T069D4 | T109A2C | T152B |
| T024 | T049A | T069E | T109A2D | T152C |
| T025 | T049A1 | T069F | T109A3 | T162 |
| T026 | T049A2 | T070 | T109A3A | T163 |
| T026A | T049A3 | T071 | T109A4 | T165 |
| T026A1 | T049A4 | T071A | T109A5 | T166 |
| T026A2 | T049A5 | T071A1? | T109A6 | T166A |
| T026A3 | T049A6 | T071B | T109A7 | T166A1 |
| T027 | T049B | T071B1 | T109B | T167 |
| T028 | T049B1 | T071B2 | T109B1 | T167A |
| T028A | T049B2 | T071B3 | T109B2 | T167B |
| T028A1 | T049B3 | T071C | T109B3 | T167C |
| T028A2 | T049B4 | T071D | T109B4 | T168 |
| T028A3 | T049B5 | T071E | T109B5 | T168A |
| T028B | T049C | T072 | T109B6 | T168A1 |
| T028B1 | T050 | T073 | T109C | T168A2 |
| T028B2 | T050A | T073A | T109C1 | T168B |
| T028C | T050B | T073A1 | T109C2 | T170 |
| T028D | T050B1 | T073A2 | T109C3 | T171 |
| T028E | T050B2 | T073A3 | T109D | T172 |
| T029 | T050C | T073B | T109D1 | T173 |
| T030 | T050D | T073C | T109D2 | T174 |
| T030A | T050E | T073C1 | T109E | T174A |
| T030B | T051 | T073C2 | T113 | T174A1 |
| T030B1 | T052 | T073D | T115 | T174A2 |
| T030B2 | T053 | T074 | T117 | T174B |
| T030B3 | T054 | T075 | T117A | T174B1 |
| T030C | T055 | T075A | T117B | T174C |
| T031 | T055A | T075B | T117B1 | T175 |
| T032 | T055B | T075B1 | T118 | T176 |
| T033 | T055B1 | T075B2 | T119 | T176A |
| T033A | T055C | T075B2A | T120 | T176B |
| T034 | T056 | T075B3 | T121 | T199 |
| T034A | T057 | T075B4 | T121A | T199A |
| T034A1 | T057A | T075C | T121A1 | T200 |
| T034A2 | T058 | T075C1 | T121A2 | T223 |
| T034B | T059 | T075C2 | T121A3 | T224 |
| T035 | T059A | T075C3 | T121B | T224A |
| T035A | T059A1 | T075C4 | T122 | T224B |
| T035A1 | T059A1A | T077 | T123 | T225 |
| T035A2 | T059A2 | T077A | T123A | T226 |
| T035A3 | T059A3 | T077B | T123B | T227 |
| T035A4 | T059A4 | T077C | T123C | T250 |
| T036 | T059A5 | T077D | T123D | T250A |
| T036A | T060 | T077E | T124 | T250B |
| T036A1 | T060A | T078 | T124A | T250C |
| T036A1A | T060B | T078A | T124A1 | T250D |
| T036A2 | T060C | T079 | T124A1A | T251 |
| T036A3 | T060D | T080 | T124A2 | T252 |
| T036A4 | T060E | T081 | T124A2A | T252A |
| T036A5 | T060F | T082 | T124A2B | T253 |
| T036B | T060G | T083 | T124A3 | T253A |

**Table S2:** ID and estimated birth years for 97 female West Coast Transient (WCT) killer whales used to estimate age of first recruitment (AFR, in years, inclusive of viable calves only) and age of first reproduction (AFRP, in years, inclusive of all calves). WCT that gave birth to a first calf in 2024 were not included in estimation of AFR, as a full year had not passed to determine whether those calves should be considered viable. A subsample of 41 females (indicated with *), which were documented at least once per year between age 10 and first recruitment, was used to calculate AFR separately and test for evidence of difference.

| **ID** | **Estimated birth year** | **AFR** | **AFRP** |
| --- | --- | --- | --- |
| T002B | 1979 | NA | 27 |
| T002C* | 1989 | 13 | 13 |
| T007B* | 1982 | 12 | 12 |
| T022 | 1977 | NA | 21 |
| T023C* | 1990 | 13 | 13 |
| T023D* | 1993 | 19 | 13 |
| T028A | 1994 | 18 | 18 |
| T028B | 1997 | 18 | 18 |
| T030B* | 1993 | 19 | 19 |
| T033 | 1972 | NA | 14 |
| T034A* | 2007 | 12 | 12 |
| T035A | 1998 | 12 | 12 |
| T036A | 1990 | 15 | 15 |
| T036A1* | 2005 | 17 | 17 |
| T036B* | 1998 | 11 | 11 |
| T037 | 1979 | 15 | 15 |
| T037A | 1994 | 13 | 13 |
| T037B* | 1998 | 14 | 14 |
| T038 | 1980 | 20 | 20 |
| T038A | 2000 | 15 | 15 |
| T038B | 2003 | 15 | 15 |
| T041A* | 1988 | 25 | 23 |
| T046B | 1988 | 15 | 15 |
| T046B1* | 2003 | 12 | 12 |
| T046B2* | 2008 | 15 | 13 |
| T046C* | 1994 | 12 | 12 |
| T049A | 1986 | 15 | 15 |
| T049B* | 1992 | 14 | 14 |
| T050 | 1980 | 15 | 15 |
| T050B | 1999 | 13 | 13 |
| T055B | 1994 | 27 | 27 |
| T059A* | 1995 | 11 | 11 |
| T059A1* | 2006 | 17 | 17 |
| T060 | 1980 | 15 | 15 |
| T064A | 1993 | 16 | 16 |
| T064B* | 2002 | 11 | 11 |
| T064B1 | 2013 | NA | 11 |
| T065A | 1986 | 13 | 13 |
| T065B* | 1993 | 18 | 18 |
| T068B | 1987 | 14 | 14 |
| T068B1 | 2001 | 12 | 12 |
| T068C | 1992 | 15 | 15 |
| T068C1 | 2007 | NA | 17 |
| T068C3 | 2012 | NA | 12 |
| T069 | 1974 | 15 | 15 |
| T069A* | 1989 | 14 | 14 |
| T069D* | 2001 | 12 | 12 |
| T071 | 1980 | 15 | 15 |
| T071B | 2000 | 13 | 13 |
| T073A | 1987 | 18 | 18 |
| T073C* | 1998 | 14 | 14 |
| T075B* | 1996 | 11 | 11 |
| T075B2 | 2015 | NA | 8 |
| T075C* | 1998 | 19 | 19 |
| T077 | 1981 | 15 | 15 |
| T078* | 1986 | 12 | 12 |
| T085 | 1977 | 15 | 15 |
| T086A* | 1988 | 13 | 13 |
| T086A1* | 2001 | 22 | 18 |
| T086A3 | 2011 | NA | 13 |
| T090* | 1980 | 20 | 20 |
| T099 | 1984 | 19 | 19 |
| T099B | 2007 | NA | 17 |
| T100B* | 1997 | 13 | 13 |
| T109A* | 1990 | 15 | 13 |
| T109A2* | 2005 | 11 | 11 |
| T109A3* | 2009 | 13 | 13 |
| T109B* | 1995 | 15 | 12 |
| T109C* | 2002 | 10 | 10 |
| T109D* | 2007 | 11 | 11 |
| T117B | 2005 | 18 | 18 |
| T121A | 1998 | 20 | 17 |
| T123 | 1985 | 15 | 15 |
| T124A* | 1984 | 12 | 12 |
| T124A1 | 1996 | NA | 28 |
| T124A2* | 2001 | 12 | 12 |
| T124A3* | 2006 | 13 | 13 |
| T124A4* | 2010 | 11 | 11 |
| T124B* | 1988 | 13 | 13 |
| T124D* | 1996 | 18 | 18 |
| T137 | 1984 | 18 | 18 |
| T141 | 1989 | 15 | 15 |
| T146 | 1987 | 12 | 12 |
| T146A | 1999 | 19 | 19 |
| T146C | 2004 | 15 | 15 |
| T151A | 2005 | 14 | 14 |
| T152 | 1995 | 17 | 17 |
| T166A | 1994 | 18 | 18 |
| T167 | 1992 | 18 | 18 |
| T168A | 1992 | 21 | 21 |
| T174A | 1998 | 17 | 17 |
| T174B | 2005 | 14 | 14 |
| T176 | 1994 | 13 | 13 |
| T199 | 1993 | 15 | 15 |
| T224 | 1998 | 15 | 15 |
| T250 | 1989 | 19 | 19 |
| T253* | 2001 | 18 | 18 |

**Table S3:** ID and estimated birth year for 98 female West Coast Transient (WCT) killer whales used to estimate mean calving interval and average annual fecundity rate. Female WCT that were born prior to the beginning of the survey (or that were physically mature at first documentation) were assigned a maximum birth year ≥10 years prior to the year in which they were first observed (Towers et al., 2019). We also separately estimated mean calving interval and annual fecundity rate from a subsample of calving intervals from 35 female WCT that were documented at least every other year beginning from age 10 (indicated with *).

| **ID** | **Estimated birth year** | **ID** | **Estimated birth year** |
| --- | --- | --- | --- |
| T002 | 1950 | T068B1 | 2001 |
| T002C* | 1989 | T068C* | 1992 |
| T007 | 1962 | T069 | 1974 |
| T007B | 1982 | T069A* | 1989 |
| T010 | ≤1964 | T069D* | 2001 |
| T012 | ≤1972 | T071 | 1980 |
| T019 | ≤1965 | T071B* | 2000 |
| T023 | ≤1964 | T073 | ≤1969 |
| T023C* | 1990 | T073A | 1987 |
| T023D* | 1993 | T073C | 1998 |
| T026A | 1990 | T075 | ≤1971 |
| T028 | ≤1972 | T075B* | 1996 |
| T028A | 1994 | T075C* | 1998 |
| T028B | 1997 | T077 | 1981 |
| T030 | ≤1967 | T085* | 1977 |
| T030B* | 1993 | T086A* | 1988 |
| T034 | ≤1969 | T090* | 1980 |
| T035A | 1998 | T099 | 1984 |
| T036 | ≤1970 | T100* | 1979 |
| T036A | 1990 | T100B* | 1997 |
| T036B* | 1998 | T101 | ≤1969 |
| T037 | 1979 | T109 | ≤1977 |
| T037A | 1994 | T109A* | 1990 |
| T037B* | 1998 | T109A2* | 2005 |
| T038 | 1980 | T109B* | 1995 |
| T038A* | 2000 | T109C | 2002 |
| T038B | 2003 | T109D* | 2007 |
| T041 | ≤1966 | T117 | ≤1976 |
| T041A* | 1988 | T121 | 1983 |
| T046 | ≤1966 | T123* | 1985 |
| T046B* | 1988 | T124 | ≤1974 |
| T046B1* | 2003 | T124A* | 1984 |
| T046C* | 1994 | T124A2* | 2001 |
| T049 | ≤1970 | T124D* | 1996 |
| T049A | 1986 | T137 | 1984 |
| T049B* | 1992 | T140 | 1983 |
| T050 | 1980 | T141 | 1989 |
| T050B | 1999 | T146 | 1987 |
| T055 | ≤1976 | T146A | 1999 |
| T059A* | 1995 | T147 | ≤1965 |
| T060 | 1980 | T151 | ≤1980 |
| T064 | ≤1971 | T152 | 1995 |
| T064A* | 1993 | T167 | 1992 |
| T064B* | 2002 | T168A | 1992 |
| T065 | ≤1968 | T174 | ≤1988 |
| T065A | 1986 | T174A | 1998 |
| T065B* | 1993 | T176 | 1994 |
| T068 | ≤1970 | T224 | 1998 |
| T068B | 1987 | T250 | 1989 |

**Table S4:** Model selection criteria for the six best candidate models examining effects of West Coast Transient (WCT) maternal age and year of calving on calving interval (CI) length. AICc: small-sample Aikaike’s Information Criterion. Only models with ΔAICc < 2 units are shown.

| **Model** | **k** | **AICc** | Δ**AICc** | **AICwt** | **loglik** | **R^2^** |
| --- | --- | --- | --- | --- | --- | --- |
| CI ~ maternal age + year* | 4 | 765.95 | 0.00 | 0.22 | -378.87 | 0.11 |
| CI ~ maternal age + year^2^ | 4 | 765.97 | 0.02 | 0.22 | -378.89 | 0.11 |
| CI ~ maternal age^2^ + year | 4 | 766.57 | 0.62 | 0.16 | -379.18 | 0.08 |
| CI ~ maternal age^2^ + year^2^ | 4 | 766.59 | 0.64 | 0.16 | -379.19 | 0.11 |
| CI ~ maternal age^3^ + year | 4 | 767.43 | 1.48 | 0.10 | -379.61 | 0.11 |
| CI ~ maternal age^3^ + year^2^ | 4 | 767.45 | 1.50 | 0.10 | -379.62 | 0.08 |

* = selected model
